# Clinical and laboratory characteristics in leptospirosis

**DOI:** 10.1186/1471-2334-14-S7-P29

**Published:** 2014-10-15

**Authors:** Raluca Elena Jipa, Nicoleta Andreescu, Eliza Manea, Sorinela Diaconu, Diana Niculescu, Doina Antonică, Nicoleta Irimescu, Ruxandra Moroti, Adriana Hristea

**Affiliations:** 1 National Institute for Infectious Diseases "Prof. Dr. Matei Balş", Bucharest, Romania; 2 Cantacuzino National Institute for Research and Development for Microbiology and Immunology, Bucharest, Romania

## Background

Leptospirosis is one of the most common zoonoses in the world, with a wide range of manifestations that can vary from mild to severe with acute hepatic and renal failure, pneumonia or meningitis. Between 2005-2011 in Romania, according to ECDC, 1740 cases have been reported. Objective: To describe clinical and laboratory characteristics of confirmed cases of leptospirosis.

## Methods

Retrospective study, between January 2004-June 2014, in one infectious diseases hospital in Bucharest. We included patients with leptospirosis diagnosis at discharge and/or positive serology for leptospirosis. Serological diagnosis of acute leptospirosis was made by microscopic agglutination test using a battery of 17 antigens from international reference strains in one national reference laboratory and/or positive IgM-enzyme-linked immunosorbent assay (ELISA). Statistical analysis was performed with SPSS v19.0; continuous variables were described with medians and ranges; categorical variables were described with numbers and percentages.

## Results

Of 132 patients with leptospirosis diagnosis at discharge, 105 (80%) had positive serology for leptospirosis. The median age was 37 (IQR 29-53) and 94 (90%) were male. 71 (68%) patients were diagnosed between May and September, 54 (51%) lived in urban areas and 8 (8%) patients had professional exposure. Leptospirosis serotype has been identified in 75 (71%) patients. 9 (9%) patients had meningeal involvement, 12 (12%) patients respiratory manifestations, 66 (63%) patients renal impairment and 38 (36%) patients coagulation impairment. The median level of alanin aminotransferase, gamma-glutamyl transpeptidase and total bilirubin were 88 IU/mL (IQR 50-158), 162 IU/mL (IQR 86-279) and 4.7 mg/dL (IQR 1.2-14.7), respectively. Leukocytosis (WBC >10.000/µL) was present in 55 (52%) patients, severe thrombocytopenia (PLT <50.000/µL) in 25 (24%) patients and 87 (83%) patients had inflammatory syndrome. In-hospital mortality was 4% (4/105).

## Conclusion

Liver and renal failure were the most common manifestations in leptospirosis. Increased awareness should be maintained in order to diagnose and initiate early adequate treatment to reduce mortality and morbidity.

